# The ESSKA-AFAS international consensus statement on peroneal tendon pathologies

**DOI:** 10.1007/s00167-018-4971-x

**Published:** 2018-05-16

**Authors:** Pim A. van Dijk, David Miller, James Calder, Christopher W. DiGiovanni, John G. Kennedy, Gino M. Kerkhoffs, Akos Kynsburtg, Daniel Havercamp, Stephane Guillo, Xavier M. Oliva, Chris J. Pearce, Helder Pereira, Pietro Spennacchio, Joanna M. Stephen, C. Niek van Dijk

**Affiliations:** 1 Academic Medical Center, University of Amsterdam, Amsterdam, The Netherlands; 2 Academic Center for Evidence based Sports medicine (ACES), Amsterdam, The Netherlands; 3 Amsterdam Collaboration on Health and Safety in Sports (ACHSS), Amsterdam, The Netherlands; 4 Fortius Clinic, London, UK; 5 Massachusetts General Hospital, Boston, USA; 6 Hospital for Special Surgery, New York, NY USA; 7 National Institute for Sports Medicine, Budapest, Hungary; 8 MC Slotervaart, Amsterdam, The Netherlands; 9 Mérignac Sport Clinic, Mérignac, France; 10 Clinica Del Remei, Barcelona, Spain; 11 Division of Foot and Ankle Surgery, National University Hospital, Singapore, Singapore; 12 Centro Hospitalar Póvoa de Varzim-Vila do Conde, Póvoa de Varzim, Portugal; 13 Ripoll y De Prado Sports Clinic: Murcia-Madrid-FIFA Medical Center of Excellence, Madrid, Spain; 14 Clinique du Sport-Centre Hospitalier, Luxembourg, Luxembourg

**Keywords:** Peroneal tendons, Tendon, Tear, Dislocation, Diagnostics, Treatment, Rehabilitation, Consensus, Guideline

## Abstract

**Introduction:**

Peroneal tendon injuries are a significant cause of lateral ankle symptoms in the active population. Accurate diagnosis and prompt treatment is important for minimizing the risk of long-term sequelae associated with chronic peroneal tendinopathy. Although several studies have been published on diagnostic strategies and treatment outcomes, there is no consensus on the optimal management of peroneal tendon pathologies.

**Purpose:**

The purpose of this ESSKA-AFAS consensus statement was to conduct an international and multidisciplinary agreed guideline on management of patients with peroneal tendon pathologies.

**Methods:**

Using the Nominal Group Technique, a panel comprised of sixteen specialists spanning nine countries was convened by the ESSKA-AFAS board. In preparation for the meeting, relevant questions were identified and supported by a systematic literature search. During the meeting, the panel members gave presentations on each question, and the evidence supporting each subject was then vetted by open discussion. Statements were thereafter adjusted on the basis of the discussion and voted upon to determine consensus using a 0–10 range Likert scale. Agreement was confirmed when a mean score of at least 7.5 was reached.

**Conclusion:**

This ESSKA-AFAS consensus statement on the optimal management of peroneal tendon pathologies is the result of international and multidisciplinary agreement combined with a systematic review of the literature.

**Level of evidence:**

V.

## Introduction

Improved knowledge based on contemporary studies has ensured that peroneal tendon disorders are a serious cause of posterolateral ankle symptoms following lateral ankle sprains (acute or chronic), despite previously being considered rare entities. Pathology may range from tendinopathy to ruptures, tears, and instability of the tendons [8, 17, 27, 33]. Since chronic peroneal tendinopathy is associated with long-term sequelae, accurate diagnosis and prompt treatment in an early stage is important.

Current practice is mainly based on level IV and V evidence. As a consequence, different diagnostic and treatment strategies are advocated in the literature without general consensus. In diagnostics, for example, different authors propose either Magnetic Resonance Imaging (MRI) or (dynamic) Ultrasound (US) as the best modality when diagnosing peroneal tendon instability [14, 34, 43]. In the treatment of irreparable peroneal tendon tears, some studies state that both tenodesis and the use of a graft are sufficient [9], while others conclude that grafting is superior to tenodesis [25].

Considering peroneal tendinopathy is associated with long-term sequelae when addressed inaccurate, adequate diagnosis and prompt treatment in an early stage is important. So far, however, no optimal management algorithm is available for diagnosing and treating different peroneal tendon pathologies. The purpose of this ESSKA-AFAS consensus meeting was to produce experience-based guidelines on the management of patients with peroneal tendon pathology, predicated on international and multidisciplinary agreement, and supported by systematic review of the literature.

## Materials and methods

This consensus statement was initiated by the Ankle and Foot Associates (ESSKA-AFAS) of the European Society of Sports traumatology, Knee surgery and Arthroscopy (ESSKA). ESSKA is one of the leading organisations worldwide concerning sports-related pathology. Using the Nominal Group Technique or mini-Delphi method [22], an international consensus panel (ICP) was selected by the board of the ESSKA-AFAS on the basis of extensive knowledge and experience regarding the management of, and science pertaining to peroneal injury. The panel was specifically compiled to gain a global representation that would cover a spectrum of opinions relevant to peroneal pathology. In total, fourteen orthopaedic surgeons, one PhD-student, and one physiotherapist were invited to join the panel. All participants were required to have at least one published or submitted peer-reviewed paper on the topic. Represented countries included Australia, France, Italy, the Netherlands, Portugal, Singapore, Spain, Sweden, the United Kingdom, and the United States of America.

### Preliminary work

After initial proposal of potential discussion topics by the board of ESSKA-AFAS, the ICP agreed upon ten final questions requiring accurate study and consensus assessment. The questions were unanimously considered to represent current controversial and relevant to daily practice topics. Each subject was designated to two independent panel members who individually performed a literature search using the PubMed and Cochrane databases to identify relevant literature published before the panel meeting date of 25th May 2017. In each case, a level of evidence was determined based on available literature, and a summary recommendation grade was then made using guidelines from the University of Oxford, Centre for Evidence-Based Medicine [10].

The following questions were considered:

1. Is there a relation between the anatomy and the development of peroneal tendon pathologies?
2. How should peroneal tendon pathologies be classified?
3. What are the key features to diagnose peroneal tendon pathology?
4. What conservative therapies may be considered and when?
5. What is the optimal treatment for peroneal tendon tears?
6. What is the optimal treatment for peroneal tendon ruptures?
7. What is the optimal treatment for acute peroneal tendon instability/dislocation?
8. What is the optimal treatment for a Painful Os Peroneum Syndrome?
9. When should hindfoot realignment procedures be considered?
10. What is the optimal post-operative protocol and rehabilitation following surgical treatment of a peroneal tendon pathology?

### Search strategy

Searching PubMed/MEDLINE and EMBASE electronic databases relevant literature was identified. Two keywords (peroneal and tendon) and related synonyms were used. All synonyms were combined with the Boolean command AND, and were linked by the Boolean command OR. The last search was performed on May 25th, 2015.

### Consensus meeting

During a two-day meeting, each of the study questions was discussed by the panel. Preceding the discussion on each question, an overview was given on the outcome of the systematic review of the literature. At the conclusion of each subject’s discussion, a level of agreement was defined based on provided recommendation. In cases where full agreement could not be reached, panel members were asked to vote using a Likert scale from 0 to 10, where 0 reflected complete disagreement and 10 complete agreement. A mean score of at least 7.5 was thereafter required to confirm consensus. When consensus was not met, the differing opinions and rationale were outlined further, and these are discussed in “Results” section.

## Results

Results of the consensus process are summarized below and are followed by a *rationale* and summary of the panel’s consensus discussion and literature review/support. For each consensus statement, the level of agreement and the level of evidence are stated in Table 1.

**Table 1:** Levels of agreement and evidence

Statement	Level of agreement	Level of evidence
1.1–1.3	Full agreement	IV
2.1–2.3	Full agreement	V
3.1–3.3	Full agreement	III
4.1–4.4	Full agreement	V
5.1–5.4	Full agreement	IV
6.1, 6.2	Full agreement	IV
7.1–7.3	Full agreement	II
7.4	6.3	II
7.5	8.0	II
8.1–8.3	Full agreement	V
9.1, 9.2	Full agreement	V
10.1–10.3	Full agreement	II

### Is there a relation between the anatomy and the development of peroneal tendon pathologies?

1.1 Several anatomical variations may predispose a patient to the development of peroneal tendon pathology.
1.2 “Overstuffing” of the peroneal tunnel is an important factor in the development of peroneal tendon pathology, and therefore, assessment of proper volume is more important than characterization of the groove shape.
1.3 Chronic loading of the tendons, as seen in a cavovarus malalignment, may predispose the tendons to pathology and this should be considered before deciding upon a treatment.

#### Rationale

The Peroneus Longus (PL) and Peroneus Brevis (PB) muscles together form the lateral compartment of the lower leg. In their distal course towards their insertion, they curl around the tip of the fibula within the superior peroneal tunnel. The panel agreed that several anatomical variabilities in the vicinity of this fibro-osseous tunnel could predispose to the development of a peroneal tendon pathology, including:

##### A low-lying muscle belly

The PL muscle becomes completely tendinous around 3–4 cm proximal to the distal fibular, whereas the PB muscle extends lower within the retromalleolar groove [31]. If the musculotendinous junction extends distal to the tip, it is considered as a low-lying muscle belly (LLMB) [11, 31]. In the literature, the relation of a LLMB to the development of peroneal tendon pathologies has been advocated. A study by Ferrecco et al. found that the distance in between the musculotendinous junction and the fibular tip was significant shorter in patients with symptomatic peroneal tendon pathology and, therefore, considered it to be a significant contributing factor [11]. Other studies, however, describe a high prevalence of LLMB also in asymptomatic cases. The panel agreed that the extent of the muscle belly does not necessarily predispose to peroneal tendon pathology, but the effect of overstuffing within the tunnel due is likely to predispose a patient to peroneal tendinopathy.

##### Accessory (peroneal) muscles

Two accessory muscles have been described within the retromalleolar groove: the peroneus quartus muscle and the peroneus quintus muscle with an incidence of 10–22% and 18–34%, respectively [47]. Both muscles can originate from the PB, the PL, the fibula, the peroneus tertius, or a combination of these structures; however, their insertion points differ. The peroneus quartus usually inserts on the extensor digitorum longus slip or along the retro trochlear tubercle of the calcaneus, while the peroneus quintus typically inserts on the dorsal aspect of the fifth metatarsal. Both accessory muscles have been linked to pain and swelling around the lateral malleolus—presumably due to over-filling of the retromalleolar peroneal tunnel as discussed above [47]. Association with other pathologies such as tendon tearing and dislocation has also been proposed in the literature, but this remains controversial [46] and the panel did not reach consensus on this topic.

##### Shape of the retromalleolar groove

At the level of the fibular tip, both tendons course through a fibro-osseous tunnel formed by the superior peroneal retinaculum (SPR) and its fibrocartilaginous ridge on the posterolateral side and the deep posterior compartment fascia and the retromalleolar groove anteromedially. As reported in current literature, the shape of the groove has been associated with peroneal tendon pathologies, with a flat or convex groove being more prone to luxation of the tendons [31]. Nevertheless, a study by Kumai et al. found that the shape is predicated more by the fibrocartilaginous ridge of the SPR than by the osseous groove [18]. Purnell et al. stated that integrity of the retinaculum is the most critical factor for preventing peroneal tendon subluxation or dislocation [26]. There was consensus among the panel about the influence of retromalleolar morphology on peroneal’ disorders.

##### Peroneal tubercle

Distal to the fibular tip, the peroneal tendons are separated by the peroneal tubercle. No clinical evidence is available on the relation between the peroneal tubercle and the development of peroneal tendon pathology. In a study by Hyer et al., the tubercle was described as prominent in 29% of cadaveric specimens and an association with pain was suggested [15]. The panel agreed that a prominent peroneal tubercle may predispose the tendons to (recurrent) tears, and excision should, therefore, be considered during treatment.

##### Os peroneum

The os peroneum (OP) is an accessory ossicle located within the distal part of the PL tendon at the level where it enters the cuboid tunnel, and protects the PL from abrasion as the tendon curls under the cuboid bone. Its incidence is estimated at 4–30% [4, 38]. Asymptomatic OPs may consists of both bony and fibrocartilaginous tissue [1, 19], whereas calcification of the OP potentially predisposes the PL tendon to tear or dislocation [38] (see section “Painful os peroneum syndrome”).

### How should peroneal tendon pathologies be classified?

2.1 The differentiation between acute and chronic peroneal pathology was not deemed to be clinically relevant, except in the case of peroneal tendon instability. Attempts at classification, therefore, should be based on the type of pathology.
2.2 Differentiation between athletes and non-athletes was determined to be an important factor in relation with treatment and outcomes.
2.3 The term “tear” usually denotes a longitudinal tear or incomplete rupture, whereas “rupture” typically denotes complete tendon discontinuity (separation of the ends).

#### Rationale

There is no consensus in the literature as to when an acute injury becomes chronic. Conflicting time frames of six weeks, three and six months have been reported. The panel concluded that the differentiation between a potentially acute or chronic injury pattern is not clinically relevant, since it does not affect treatment; the panel did agree, however, that this differentiation might influence both outcome and prognosis, since acute injuries have a better healing tendency. Concerning peroneal tendon instability, the panel concluded that it is important to differentiate between acute and chronic, since management does differ between the two groups (see section  “What is the optimal treatment for acute peroneal tendon instability/dislocation?”). Treatment and outcome may also be determined by whether the injury is sustained in an athlete rather than a non-athlete.

The panel agreed that peroneal tendon pathology is best classified by type of pathology, as divided into three categories: tendinopathy, tear/rupture, or instability/dislocation. Tears are classified as either a partial (simple or complex) longitudinal tendon tear, that does not result in complete discontinuity of the muscle tendon unit, or a rupture including a transverse discontinuity and resulting in complete dissociation between muscle and tendon at that level.

### What are the key features to diagnose peroneal tendon pathology?

**Fig. 1:**
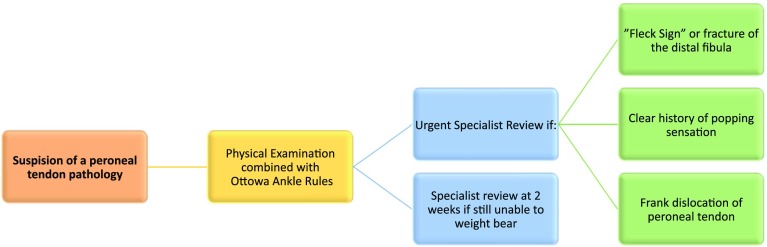
Diagnostics

3.1 Initial assessment of a patient presenting with an acute ankle injury should follow the Ottawa ankle guidelines.
3.2 Based on imaging and physical examination, a specialist should be consulted for further examination. Both US and MRI are appropriate imaging modalities for the evaluation of peroneal tendons.
3.3 Peroneal tendoscopy should be reserved for patients with high clinical suspicion of peroneal tendon pathology based on history and clinical exam, but with the absence of any positive findings on imaging. See Fig. 1 for a schematic algorithm on diagnostic management of peroneal tendon pathology.

#### Rationale

Pathology of the peroneal tendons may present with a broad variety of symptoms. Acute dislocations typically present following an identifiable injury in a previously asymptomatic ankle [28]. On investigation, there is posterolateral swelling and tenderness specifically over the SPR [12]. Dislocation can frequently be reproduced on resisted eversion of the ankle.

Acute tears are likely to present with a sudden onset of pain and swelling, which also might be caused by the additional pathology such as a lateral ligament rupture often accompanying the acute pathology. Acute injuries present with bogginess and tenderness to palpation around the distal fibula [36]. PB tears usually present with pain around the distal fibula, whereas PL tears typically present with pain near the peroneal tubercle and cuboid tunnel. Examination may also reveal respective weakness during ankle eversion and first ray plantarflexion [5].

When assessing a patient presenting with an acute ankle injury, the panel agreed that initial assessment should follow the Ottawa ankle guidelines proposed by Steil et al [39], including anteroposterior and lateral weight-bearing radiographs of the affected ankle and, if foot pathology is suspected, an oblique view. Review by a specialist is reserved for cases where imaging reveals a “fleck sign” or fracture of the distal fibula, or in case of a clear history of a “popping” sensation or frank dislocation of the tendons(s). In addition, if the patient is unable to weight bear by one to two weeks postinjury, referral to the specialist is warranted. The panel agreed that specialist evaluation should also consider and evaluate for other causes for lateral ankle pain.

Both MRI and  US are appropriate investigations and the choice is dependent on the clinician’s preference, user expertise, and the availability of the imaging modality. Tendoscopy may be beneficial when there is a high clinical suspicion of peroneal tendon pathology in the absence positive findings on imaging [16].

### What conservative therapies may be considered and in case of which pathology?

**Fig. 2:**
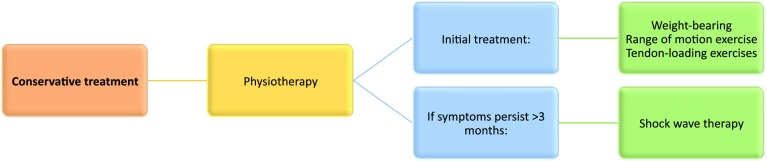
Conservative treatment

4.1 Conservative management should be considered in all patients with a peroneal tendon pathology.
4.2 In the acute situation, conservative treatment should concentrate on additional pathology such as a lateral ligament rupture. Treatment includes ice, compression, and elevation. Range of motion and exercises should be started when clinically relevant.
4.3 Shockwave therapy should be considered when initial measures fail.
4.4 The use of platelet-rich plasma is not supported by the literature to approve its use. Figure 2 presents a schematic algorithm on conservative treatment of acute peroneal tendon pathologies.

### Rationale

There is broad agreement among the panel and in the literature that under the vast majority of circumstances, conservative management of acute peroneal tendon pathology is treatment of choice. Nonetheless, there is limited and varied evidence on the outcomes of conservative treatment in acute peroneal tendinopathy [6, 21, 35]. Initial treatment is directed towards additional pathology and consists of rest, ice, compression, and elevation. When painless, the patient may start weight bearing followed by range of motion and tendon-loading exercises. The panel agreed that immobilization should be avoided.

When symptoms persist beyond three months, there is some suggestion for the use of shockwave therapy in tendinopathy of the lower extremity [20, 42]. The panel supported this application under those circumstances. With regard to the use of platelet-rich plasma, the panel agreed that at this time, there is insufficient evidence to support its use in the treatment of peroneal tendinopathy [6].

### What is the optimal treatment for peroneal tendon tears?

**Fig. 3:**
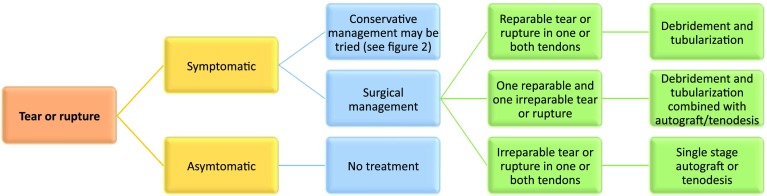
Treatment of tears and ruptures

5.1 Treatment should be reserved for symptomatic patients only.
5.2 Initial management consists of conservative treatment.
5.3 Concerning operative management, the first choice of treatment includes debridement and repair/tubularization of one or both tendons as indicated. When such treatment is not feasible, single-stage autograft with the hamstrings, or side-to-side tenodesis are recommended. When one of the two tendons is deemed irreparable, perform debridement and tubularization on the reparable tendon and use autograft or tenodesis to treat the irreparable tendon. In cases when neither tendon can be repaired nor the proximal muscle tissue is healthy, single-stage autograft is recommended. Whenever possible, grafting is preferred over tenodesis.
5.4 In tenodesis, there is no preference of PB to PL or PL to PB. In Fig. 3, a schematic algorithm for the treatment of peroneal tendon tears is presented.

#### Rationale

The prevalence of peroneal tendon tears in general population remains unknown, but cadaveric studies found tears in 11–38% of the studies ankles [23, 37]. It is assumed that only a percentage of all peroneal tendon tears becomes symptomatic and the panel, therefore, agreed that surgical treatment should only be performed in symptomatic patients. Different treatment algorithms have been proposed in the literature [9, 17, 27], suggesting that if less than 50% of the cross-sectional area of the tendon is involved in the tear, then any affected tissue can be debrided and tubularized. This 50% threshold, however, remains quite arbitrary and is not based on any substantiated data. The panel decided that it is always preferable to attempt to preserve the tendon(s) and, therefore, agreed that primary debridement and tubularization should always be tried when there can be at least some reasonable native tendon left behind in the repair (resistant to surgeon’s manual pull stress), even if less than 50%. In the literature, treatment of peroneal tendon tears with primary debridement and repair has been associated with excellent return to full activity and patient-reported outcome scores [7, 27, 32].

In cases where repair of one or both tears is not possible, the panel recommends single-stage grafting. Autograft is preferred over allograft because of both its mechanical and biological characteristics. Concerns associated with the use of an allograft include tissue availability, delayed graft incorporation, strength, disease transmission, and fatigue (creep) [41].

The panel favours grafting over a tenodesis procedure, mainly because tenodesis directly affects biomechanical balance of the foot. A cadaveric study by Pellegrini et al. found insufficient tension on the peroneal tendons after tenodesis of the PB to the PL, while an allograft was associated with substantial restoration of the tension [25]. In cases where performing tenodesis is indicated, therefore, it seems that PL to PB transfer would be the better option and transfer of the PB to the PL should be avoided. The panel does not recommend a tendon transfer using the flexor digitorum longus or flexor hallucis longus, because the procedure has several biomechanical limitations and is associated with significant deficits in strength and balance on the longer term [33].

The panel agreed that predisposing abnormalities possible contributing to the development of peroneal tendon tears should be treated simultaneously with the tear.

Examples include a hypertrophic peroneal tubercle, a LLMB or bulky PB muscle belly, peroneal subluxation or dislocation, or an accessory tendon. When left untreated, any of these may lead to recurrent tearing, persistent pain, and dysfunction [5, 24].

### What is the optimal treatment for peroneal tendon ruptures?

6.1 Complete rupture of one tendon can be treated conservatively in the inactive and asymptomatic patient.
6.2 In active patients, symptomatic complete rupture of one of the two peroneal tendons should usually be treated with repair. If repair is not possible, a single-stage hamstring autograft or tenodesis may be performed. In tenodesis, there is no preference of PB to PL or PL to PB. When these options are not feasible, FHL or FDL tendon transfer is a final option. In Fig. 3, a schematic algorithm for the treatment of peroneal tendon ruptures is presented.

#### Rationale

Complete rupture of one of the tendons can be treated conservatively in the event that the patient remains low demand and asymptomatic. In the symptomatic or highly active patient, however, surgical management is often required to support return to sports. Patients with rupture of both tendons benefit from surgical management to treat their symptoms. The panel agrees that, if possible, the tendon tissue should be preserved and, therefore, recommends end-to-end repair of the rupture(s).

In cases when this is not possible, the panel recommended the same treatment algorithm agreed for peroneal tendon tears. If grafting or tenodesis remains insufficient, a tendon transfer may be considered [33]. It should be recognised that elite athletes may not return to their pre-operative level of sports after surgical treatment for peroneal tendon rupture.

### What is the optimal treatment for acute peroneal tendon dislocation?

**Fig. 4:**
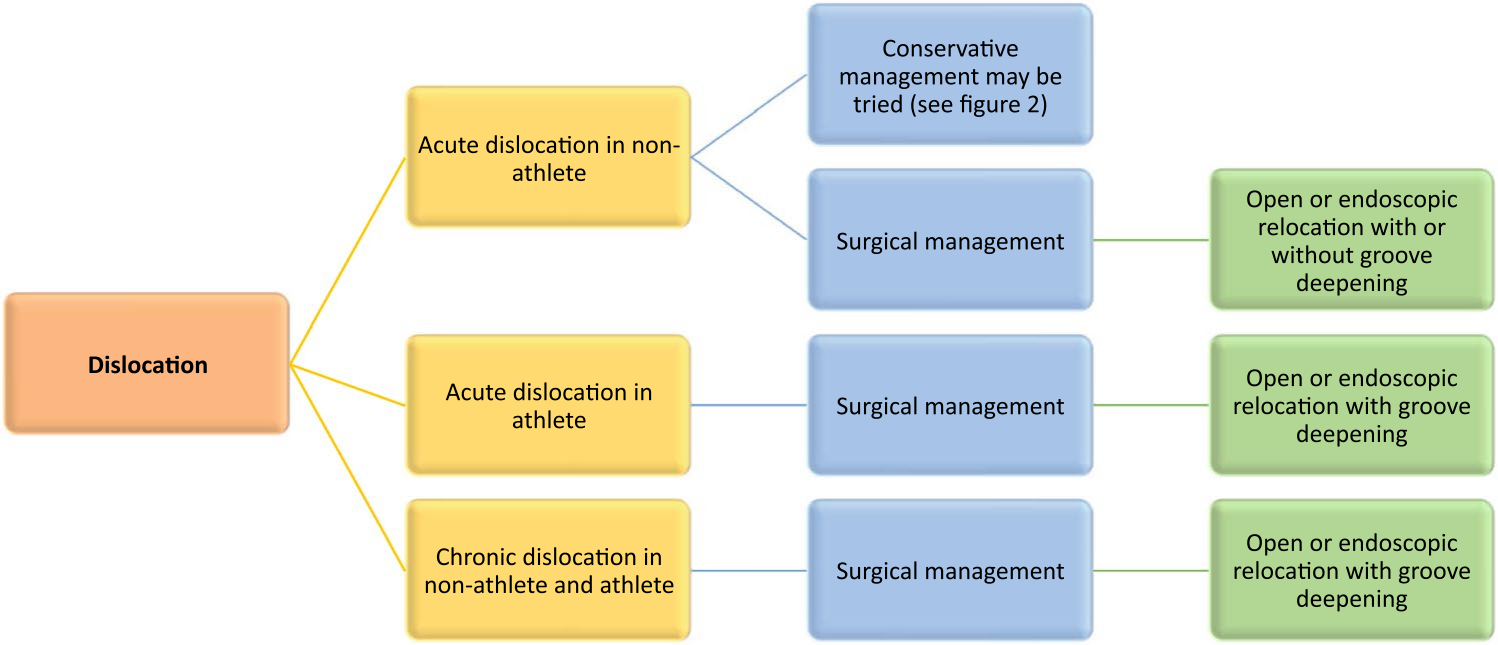
Treatment of dislocation

7.1 Treatment of peroneal tendon dislocation should be based on whether it is an acute or chronic injury and whether or not the patient is an athlete.
7.2 The non-athlete with an acute dislocation may be offered conservative management but should be warned that there is a 50% chance of recurrent dislocation. In case of unsuccessful conservative management or chronic instability, surgical intervention is advised.
7.3 Surgery is recommended for elite athletes having sustained either acute or chronic dislocation.
7.4 Surgery in non-athletes with acute peroneal instability consists of reduction of the tendons into the retrofibular groove and repair of the SPR. There was no agreement as to whether to perform an additional groove deepening in non-athletes.
7.5 There was agreement that surgical treatment in athletes should routinely include groove deepening, regardless of other possible treatment gestures. Figure 4 shows a schematic algorithm on treatment of peroneal tendon dislocation.

#### Rationale

Acute peroneal tendon dislocation typically occurs after a forced eccentric contraction of the peroneal muscles combined with dorsiflexion and eversion of the ankle [29]. Multiple management options have been proposed for the treatment of peroneal dislocations, generally aiming to repair or reconstruct the SPR, correct predisposing factors and increase the volume of the peroneal tunnel. While the benefits of surgery have been shown in the literature [44], the value of conservative management remains unclear. The current evidence is limited to a number of case reports and small retrospective series suggesting that the risk of recurrent peroneal instability is approximately 50% [21]. As discussed in the section “Classification and Terminology”, the panel determined that choosing optimal treatment necessitates differentiation between acute and chronic injury and between the athlete and non-athlete population.

For acute instability in non-athletes, the panel agreed that both conservative and surgical management are indicated. Although conservative management carries a 50% risk of failure, secondary surgical treatment does not lead to a worse prognosis or alter the surgical options available if it fails. Conservative management should include immobilization in a cast in slight plantarflexion or in a boot with a 2 cm heel wedge for six weeks. If, however, the patient has a suspected or confirmed anterior talofibular ligament injury, they should be immobilized in a neutral position to not compromise the lateral ligament healing. Physical therapy is commenced after six weeks with peroneal strengthening and ankle proprioception exercise.

Surgery in non-athletes with acute peroneal instability consists of reduction of the tendons into the retrofibular groove and repair of the SPR. There was no consensus as to whether an additional groove deepening procedure was required in open repairs. In addition, no agreement was reached as to whether endoscopic or open treatment was favoured, but it was agreed that either was acceptable with the acknowledgement that endoscopic treatment may have less potential complications and allows for earlier functional rehabilitation. If endoscopic stabilization is performed, the panel agreed that the most appropriate technique is to debride the lateral edge of the fibula, where the retinaculum has been lifted away, followed by groove deepening. The SPR does not require formal repair; however, this option is valid.

In the athlete with acute instability, conservative management is not advised and early surgical stabilization is the treatment of choice. Opposing to the non-athlete population, the panel agreed that, for this group, surgery should include deepening of the retromalleolar groove. There was agreement that both endoscopic and open treatment are accurate surgical modalities. As stated above, however, endoscopic treatment may allow earlier functional rehabilitation, which may allow earlier return to play.

In chronic injuries, the panel recommended surgical stabilization as the first line treatment with deepening of the retromalleolar groove. In chronic injuries, shortening of the tendons is often seen and groove deepening allows for accommodation of this and greater stability. There was no favour as to the choice of endoscopic or open treatment.

In all types of peroneal instability, there was agreement that in open stabilization, the SPR should always be repaired, but extra care should be taken not to over tighten the SPR, which could result in stenosis of the retromalleolar space. It was also recommended to treat potential tunnel overcrowding factors such as a LLMB or an accessory muscle.

### What is the optimal treatment for a painful OP syndrome?

**Fig. 5:**
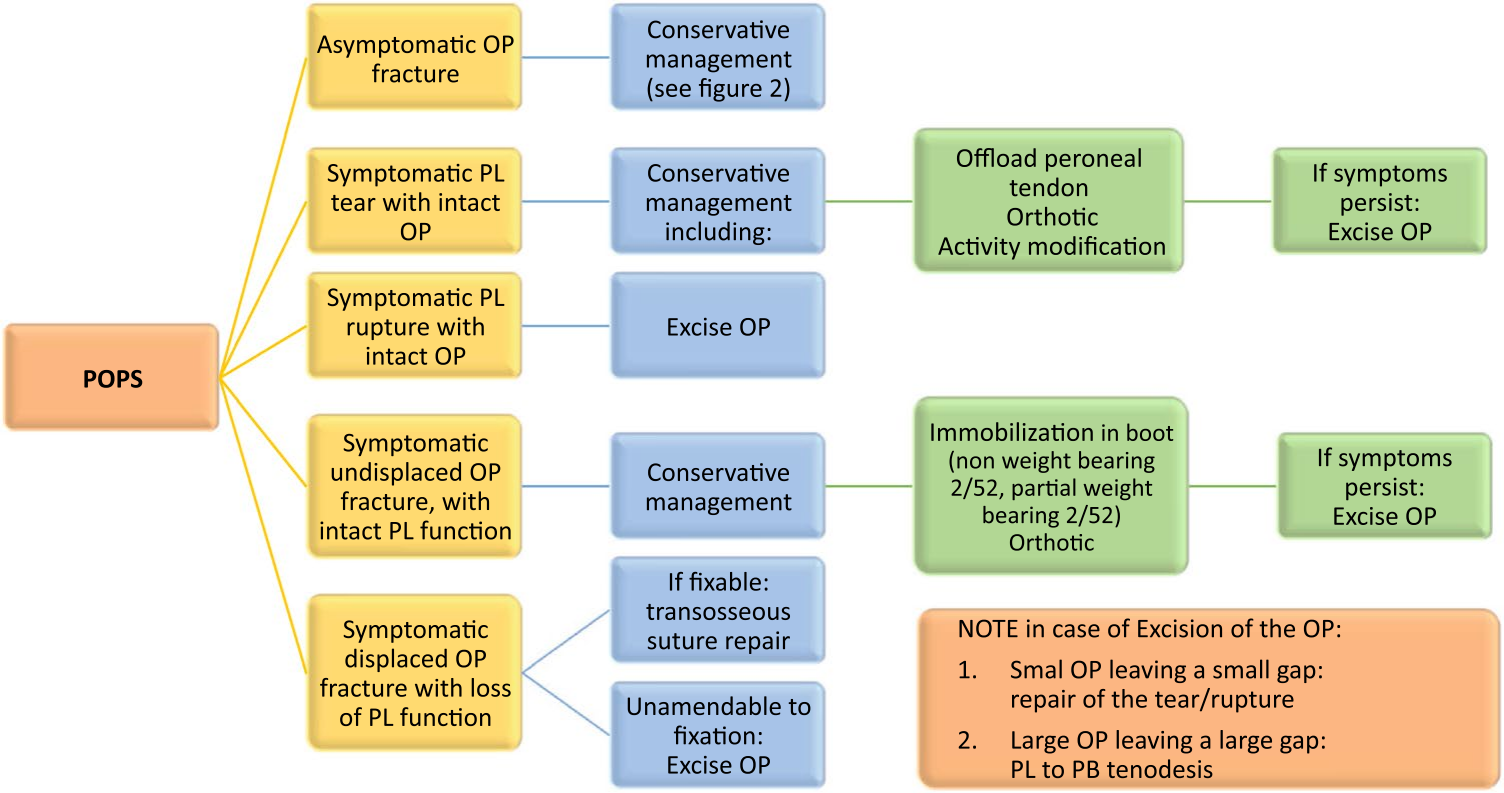
Treatment of the painful OP syndrome. *POPS* Painful Os Peroneum syndrome, *OP* Os peroneum, *PL* peroneus longus tendon

**Fig. 6:**
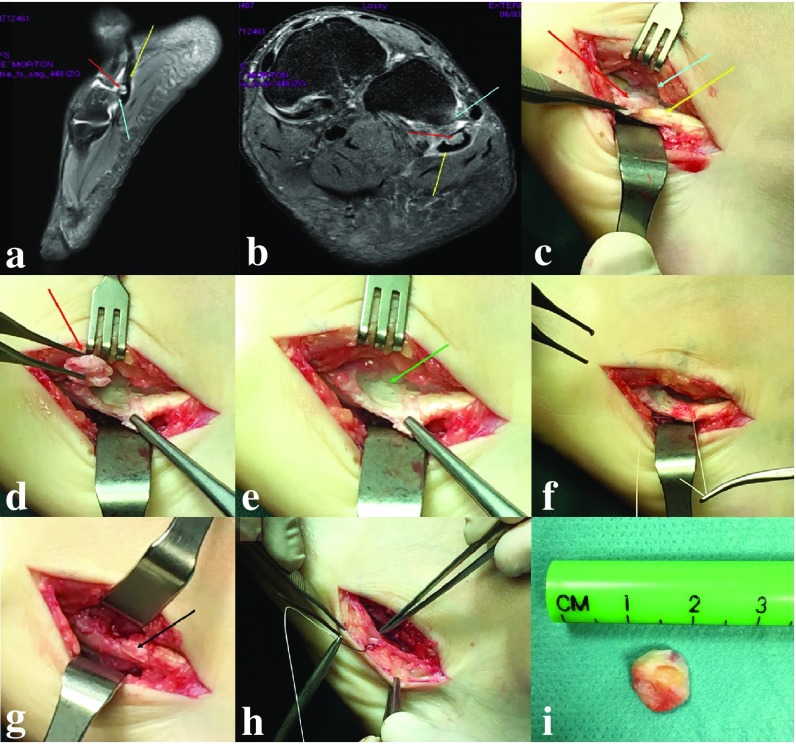
Case of symptomatic Os Peroneum.* Peroneum* causing impingement with the cuboid. *Os Peroneum* (OP—red arrows); *Peroneus Longus* (PL—yellow arrows) on lateral (**a**) and axial (**b**) MRI views. In the conflict area of the OP with the cuboid it is visible some bone edema in T2 MRI sequences (light blue arrows). **c** PeroperaCve image with visibility of the OP (red arrow), PL (yellow arrow) and impingement area with the cuboid (light blue arrow). The OP is detached from the PL keeping the integrity of the PL. The peroneal Cssue is flaIened (green arrow) in the zone where the OP was removed (**e**). **f** Reinforcement sutures of the PL are performed with tubularizaCon of the flaIened area (**g**—black arrow). **h** Be aware of the close connecCon with the sural nerve (pointed by surgical tweezers) during all the procedure and confirm its integrity in the end before closure of the wound. **i** OP after removal in one piece

8.1 Patients with tears of the PL, associated with an OP and in the absence of frank rupture, should be initially treated conservatively.
8.2 Symptomatic rupture of the PL tendon or symptomatic OP syndrome that fails conservative management should be treated surgically. If the PL cannot be directly repaired, then it can either be tenodesed to the PB tendon or an allograft interposition graft can be used.
8.3 Fractures of the OP can either be repaired or excised and treated as per a PL rupture. A schematic algorithm on optimal management is shown in Fig. 5. A rare case of a fractures OP is presented in Fig. 6.

#### Rationale

The painful OP syndrome (POPS) is a relatively uncommon condition that covers a broad spectrum of pathology, including acute and chronic fracture or diastasis of the OP, a tear of the PL, a frank avulsion rupture, an enlarged peroneal tubercle which entraps the OP and PL, or a tenosynovitis without rupture or tear [38]. Currently, there is only level IV and V evidence on the treatment of POPS [40].

The panel agreed that the treatment regime should be based on the presenting symptoms and the diagnosed pathology. Symptomatic tears without frank rupture of the PL with intact power should be treated conservatively with measures to offload the tendons and provide symptomatic relief, for example, with orthotics and activity modification. There was no consensus on the use of corticosteroids injections as a therapeutic or diagnostic tool and the benefits need to be balanced with the risk of a tendon rupture, because there is currently insufficient evidence available to draw any meaningful conclusions. It was acknowledged by the panel that the reported risk of complete rupture following US-guided injection of corticosteroid into the peroneal tendon sheath is actually low.

The panel agreed that in symptomatic ruptures of the PL with loss of function, the decision for operative intervention should be based on the patient demands. In addition, the group remarked that the consequences of a loss in PL function have not been clearly defined in the literature. In addition, the panel agreed based on anatomical studies that it is important to consider the presence of a fibrocartilaginous OP even if there is a rupture of the PL without X-ray evidence of an OP.

If surgery is indicated, a direct repair is recommended combined with excision of any OP present. If a direct repair cannot be obtained, either a PL to PB tenodesis or repair with the use an interposition graft can be performed. If there is a displaced fracture of the OP leading to loss of PL function, there is mixed evidence for either repair of the osseous OP or excision and direct repair of the tendon [2, 30, 38, 40]. Express concern with osseous repair is being able to obtain adequate stability and delayed union if the etiology is a stress fracture. Controversially, excision of the OP may leave a large defect affecting the ability to perform a direct repair of the rupture. The panel agreed that it is the surgeon’s preference to either perform a PL to PB tenodesis or interposition graft. It was acknowledged that after complete PL rupture near the cuboid, direct repair (e.g., osseous tunnel, suture anchors) or interposition graft is technically very difficult and tenodesis of PL to PB may be the most practical option.

In rare cases of an undisplaced fracture of the OP with intact PL function, then, the panel agreed that this can be treated conservatively with boot immobilization, non-weight bearing for two weeks, partial weight bearing for two weeks, and on-going orthotics to offload the PL.

### When should hindfoot realignment procedures be considered?

9.1 Hindfoot realignment procedures are recommended only for patients with hindfoot deformity, such as varus or valgus, associated with joint degeneration or instability.
9.2 Care should be taken when performing these procedures in elite athletes, once they might be less likely to return to their pre-operative level of sports after surgical realignment of the hindfoot.

#### Rationale

Peroneal tendon pathology is often seen with both cavovarus and planovalgus deformity, predisposing these tendons to compression or overuse injuries within the sub-fibular region [36]. The etiology of cavovarus deformity is multifactorial, but is most commonly due to a muscle imbalance in the lower leg and foot. The PL insertion on the plantar aspect of the first metatarsal has been postulated as a cause of deformity in the cavovarus foot [3, 4]. Indeed, Helliwel et al. demonstrated that in 75% of cavovarus feet, the PL is enlarged on MRI [13]. In addition, Redfern et al. found that in patients presenting with a peroneal tendon tear, 32% had a concomitant isolated hindfoot varus or cavovarus foot deformity [27].

Currently, there is no evidence on the isolated effect of a calcaneal osteotomy in peroneal tendon injury. Some case studies support the role of a calcaneal osteotomy for peroneal tendon pathology with a cavovarus deformity [3, 27]. The panel agreed that hindfoot realignment procedures should be reserved for symptomatic varus or valgus associated joint degeneration and/or ankle instability and not in the case of an isolated peroneal pathology.

The panel agreed that in athletes with hindfoot malalignment and peroneal tendon pathology, correction of the hindfoot malalignment is probably best avoided. The panel’s experience is that it is not uncommon for athletes to have asymptomatic idiopathic hindfoot varus and in case when this is corrected, the biomechanical change in the lower limb alignment may have a detrimental effect on their level of elite performance.

### What is the optimal post-operative protocol and rehabilitation following surgical treatment of peroneal tendon pathology?

**Fig. 7:**
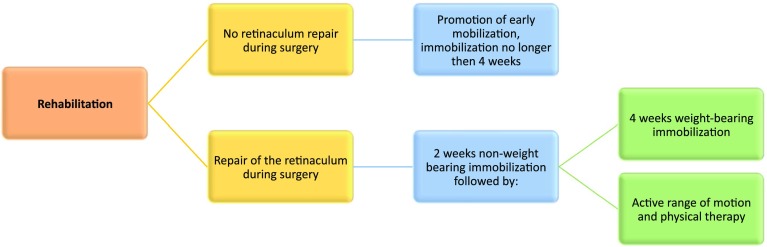
Post-treatment rehabilitation

10.1 For optimal rehabilitation, one must distinguish whether or not the SPR was repaired during the surgical procedure.
10.2 When the SPR is not repaired, rehabilitation should be goal- and not time-based with the promotion of early mobilization.
10.3 When surgery included repair of the SPR, rehabilitation should consist of two-week non-weight bearing in a lower leg cast, followed by four weeks of weight bearing in a cast or a walker boot. At two weeks post-operatively, active range of motion and physical therapy should be encouraged. The tendons should not be loaded until six weeks post-operatively. In addition, several pre-operative sessions are recommended for best achievement of rehabilitation objectives, although these may not be feasible. Figure 7 shows a schematic algorithm on post-operative management.

#### Rationale

A broad range of rehabilitation protocols has been described without enough scientific support to enable proposing any evidence-based post-operative protocol [45]. Based on a recent review by van Dijk et al., presenting an overview of all different protocols being used [45], the panel agreed that it is mandatory to distinguish whether or not the SPR was repaired during the surgical procedure. In cases where the SPR was not repaired, but the stabilization of the tendons relied on the groove deepening alone, the immobilization time should be minimalized to prevent tethering of the tendon(s). It is, therefore, recommended to aim for an immobilization period no longer then four weeks. The panel agreed that in the future, this period of protection might be shortened.

When the SPR is formally repaired, a minimum immobilization time of 6 weeks is important for sufficient healing of the retinaculum. The initial two-weeks non-weight bearing is advised. After these two weeks, the patient is allowed weight-bearing immobilization combined with physiotherapy and supervised range of motion to allow peroneal movement while protecting the repaired SPR. For optimal healing, pain free loading of the peroneal tendons should not be performed until six-weeks post-operative. The panel agreed that commencement of running activities should not be based on time criteria, but rather be dependent upon the patient’s pre-operative condition, the ability to perform a single heel rise, and the patient’s overall strength, neuromuscular control, and proprioceptive ability.

## Conclusion

Considering the scarce published knowledge, this consensus statement on peroneal tendon pathology summarizes the most practical and scientifically supported diagnostic and treatment algorithms for enabling optimized management of peroneal tendon pathology. The guidelines are based on international and multidisciplinary expert agreement following the Nominal Group Technique, combined with a systematic review of available literature.
